# A highly sensitive screening system to evaluate the reversibility of neuroendocrine prostate cancer to prostate adenocarcinoma

**DOI:** 10.1002/cam4.70047

**Published:** 2025-02-27

**Authors:** Tomohiro Fukui, Kosuke Okasho, Yukiko Okuno, Maki Fujiwara, Kensuke Hikami, Arinobu Fukunaga, Takuro Sunada, Yuki Kita, Takayuki Sumiyoshi, Takayuki Goto, Ryoichi Saito, Osamu Ogawa, Takashi Kobayashi, Shusuke Akamatsu

**Affiliations:** ^1^ Department of Urology Kyoto University Graduate School of Medicine Kyoto Japan; ^2^ Medical Research Support Center, Graduate School of Medicine Kyoto University Kyoto Japan; ^3^ Department of Urology Nagoya University Graduate School of Medicine Nagoya Japan

## Abstract

**Background:**

Lineage plasticity in prostate cancer primarily induces transdifferentiation of adenocarcinoma into neuroendocrine prostate cancer (NEPC). Lineage alteration is largely driven by epigenome and is potentially reversible. Nonetheless, evidence supporting NEPC reversibility is lacking in materials derived from clinical specimens. Hence, we established KUCaP13, a novel NEPC cell line derived from the tissue of a patient initially diagnosed with adenocarcinoma, which later recurred as NEPC. We aimed to prove the reversibility of cellular lineage by high‐throughput chemical screening using KUCaP13 cells.

**Methods:**

Compounds responsible for androgen receptor (AR) re‐expression in KUCaP13 cells were screened. A reporter gene, androgen response element luciferase (AREluc), was transduced into KUCaP13 cells to detect AR activity using luciferase assay.

**Results:**

Positive control cells (KUCaP13_AREluc_AR) overexpressing AR showed enhanced luminescence upon administration of synthetic androgen. An initial screen of 1552 compounds revealed 30 candidate molecules potentially enhancing luciferase luminescence. In the second screening, we eliminated false positives and validated the findings using luciferase assay and quantitative real‐time polymerase chain reaction. However, all hit compounds were confirmed as false positives, probably due to their inhibitory activity on luciferase.

**Conclusions:**

We developed a highly sensitive screening system to evaluate the reversibility of plasticity in NEPC using a novel cell line. However, no compound demonstrated the ability to re‐express AR in this cell line. Nevertheless, in future studies, this screening system could prove valuable for elucidating the mechanism of lineage plasticity in NEPC and for developing novel therapies focused on reversing plasticity.

We established a robust and sensitive androgen response element luciferase reporter assay to monitor androgen receptor (AR) activity using KUCaP13 cells, a novel human‐derived treatment‐related neuroendocrine prostate cancer (t‐NEPC) cell line. A high‐throughput screening using a chemical library to identify potential compounds that induce AR re‐expression in KUCaP13 cells revealed 30 candidate molecules potentially enhancing luciferase luminescence; however, subsequent validation steps demonstrated these signals to be false‐positives. Despite not achieving the goal of AR re‐expression, this study stands as a significant proof‐of‐concept for the application of high‐throughput screening approaches in t‐NEPC research.

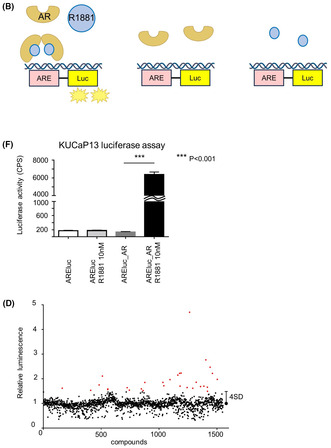

## 1 INTRODUCTION

Prostate cancer grows in an androgen‐dependent manner, and the standard therapy for advanced prostate cancer is endocrine therapy targeting the androgen receptor (AR) signaling pathway. However, with the widespread use of potent next‐generation AR signaling inhibitors (ARSIs), the incidence of treatment‐related neuroendocrine prostate cancer (t‐NEPC), which is completely independent of the AR pathway, is rapidly rising.^1^, ^2^ Unlike its AR‐dependent ancestor, t‐NEPC remains a poor prognosis cancer, lacking effective treatment options, and thus necessitating the exploration and development of innovative therapies.^2^

Transdifferentiation of prostate adenocarcinoma into neuroendocrine prostate cancer (NEPC) occurs through lineage plasticity.^3^, ^4^, ^5^ Lineage plasticity is a biological process that enhances cell survival by enabling adaptation to the environment, avoidance of stress, or tissue repair.^4^ Within cancer, lineage plasticity facilitates the development of therapy resistance in cancer cells by reprogramming into therapy‐resistant phenotypes that bypass targeted therapies.^5^ This phenomenon is especially notable in cancer varieties where there are potent targeted therapies for key growth pathways, such as AR‐driven prostate cancer, epidermal growth factor receptor (EGFR)‐mutant lung cancer, and BRAF‐mutant melanoma.^4^

Recent genomic analyses have revealed several alterations enriched in t‐NEPC, with the loss of tumor suppressor genes (*TP53* and *RB1*) being crucial genomic changes linked to t‐NEPC.^3^, ^6^ Moreover, epigenetic genes, such as *EZH2* and *SOX2*, induce neuroendocrine (NE) transdifferentiation.^7^, ^8^ A study with gene‐engineered mice lacking *Pten* and *Rb1*, or all three (*Pten*, *Rb1*, and *Trp53*), showed reduced expression of AR and increased expression of NE‐related genes, phenocopying human NEPC. Furthermore, using EZH2 inhibitors restored AR expression and sensitivity to antiandrogen therapy.^7^ These findings suggest that plasticity in t‐NEPC is potentially reversible, and regulating cellular lineage could serve as a novel therapeutic strategy. However, EZH2 inhibition did not increase AR expression or activity in organoids from human NEPC.^9^ To date, the reversibility of lineage plasticity in prostate cancer has not been confirmed in human‐derived t‐NEPC clinical models.

One major hurdle in NEPC research is the paucity of human‐derived t‐NEPC cell lines suitable for genetic manipulation or large‐scale compound screening. Previously, we created a novel t‐NEPC cell line called KUCaP13, derived from a patient‐derived xenograft (PDX) and verified its lineage originating from prostate adenocarcinoma.^10^ The cell line's origin as prostate adenocarcinoma is supported by the preservation of a homozygous *CHD1* deletion from patient tissue to the cell line.^10^ Concurrent inactivation of *TP53*, *RB1*, and *PTEN* in KUCaP13 makes it an ideal candidate for NEPC research.^11^ KUCaP13 cells grow suspended in culture, forming spheroids. They are dissociated into single cells to facilitate lentiviral transfection, fluorescence‐activated cell sorting (FACS) sorting, and accurate cell counting. Herein, we hypothesized that t‐NEPC can be transdifferentiated back to adenocarcinoma and attempted to prove the reversibility of cellular lineage by compound screening using KUCaP13 cells (Figure S1). To this end, we developed a highly sensitive screening system to detect AR re‐expression in KUCaP13 cells and performed a high‐throughput screening using a chemical library.

## 2 MATERIALS AND METHODS

### 2.1 Cell culture

The LNCaP cell line was purchased from the American Type Culture Collection (Rockville, MD, USA). KUCaP13 and LNCaP cells were cultured in RPMI 1640 medium (Nacalai Tesque, Kyoto, Japan; Cat no. 30264‐56) supplemented with 10% fetal bovine serum (Merck Millipore, Darmstadt, Germany), 12.5 mmol/L HEPES, penicillin, and streptomycin, at 37°C in 5% CO_2_. For experiments investigating AR activity and expression, the cells were grown in an androgen‐free medium composed of phenol red‐free RPMI 1640 (Thermo Fisher Scientific, Waltham, MA, USA; Cat no. 11835030) containing 10% charcoal‐stripped fetal bovine serum (Cytiva, Tokyo, Japan; Cat no. SH30068.03) and 12.5 mmol/L HEPES, with or without R1881 (Merck Millipore).

### 2.2 Lentiviral transduction of cell lines

Androgen response elements (AREs) with luciferase reporter was introduced into LNCaP cells to generate LNCaP_AREluc cells using AR‐Luciferase (Puro) lentiviral particles (LVP914‐P) (GenTarget Inc, San Diego, CA, USA). As a negative control, LNCaP was infected with miniPro (Null)‐Luc (Puro) lentiviral particles (Path‐Ctr3) (GenTarget Inc) to generate LNCaP_miniluc cells using polybrene. The infected cells were then selected using puromycin (1.0 μg/mL).

To facilitate transduction with lentivirus, KUCaP13 cells were dissociated into single cells using TrypLE™ Express (Thermo Fisher Scientific) for 10 min at 37°C on a shaking platform (1200 rpm). Further, KUCaP13 cells were infected with AR‐Luciferase (GFP) lentiviral particles (LVP914‐G) (GenTarget Inc) using LentiBlast Premium (OZ Biosciences, Marseille, France) to generate KUCaP13_AREluc cells. GFP‐positive cells were isolated utilizing BD FACS AriaTM II (BD Biosciences, San Jose, CA, USA) and fluorescence images were captured with a BZ‐8000 microscope (KEYENCE, Osaka, Japan). Plasmid pLENTI6.3/AR‐GC‐E2325 (Plasmid #85128) was purchased from Addgene (Watertown, MA, USA) for AR overexpression, and viral particles were produced as described previously.^10^ KUCaP13_AREluc cells were infected with the viral particles to generate KUCaP13_AREluc_AR cells, which overexpresses AR. The infected cells were subjected to selection with blasticidin (4.0 μg/mL).

### 2.3 Western blotting

We performed protein extraction from cell pellets and measured protein concentration as previously described.^10^ Samples containing 12.5 μg of protein underwent loading, separation through sodium dodecyl‐sulfate polyacrylamide gel electrophoresis, and subsequent transfer onto polyvinylidene fluoride (PVDF) membranes (Immobilon‐P PVDF 0.45 μm, Merck Millipore; Cat no. IPVH304F0) using a Mini Trans‐Blot Cell system (Bio‐Rad Laboratories, Hercules, CA, USA). After blocking in 5% milk in TBST buffer for 1 h at room temperature, the membrane was subsequently incubated overnight at 4°C with primary antibodies against AR (Cat no. 5153) and α‐tubulin (Cat no. 2144), both sourced from Cell Signaling Technology (Danvers, MA, USA). Following three washes with 1 × TBST buffer, the membrane was incubated with a secondary antibody for 1 h at room temperature. Protein bands were then detected using Pierce™ ECL Western Blotting Substrate (Thermo Fisher Scientific. Cat no. 32106) and photographed with ImageQuant LAS 4000 mini (Fuji Film, Tokyo, Japan).

### 2.4 Luminescence measurements

Luciferase assays were performed in white 96‐well plates seeded with AREluc‐transfected cells. The firefly luciferase activities were quantified using a ONE‐Glo™ EX Luciferase Assay System (Promega, Madison, WI, USA). Luminescence was measured using an ARVO X5 (PerkinElmer, Waltham, MA, USA) luminometer.

### 2.5 Chemical library

The chemical library used for chemical screening were provided by the Medical Research Support Center, Graduate School of Medicine, Kyoto University. The chemical library comprised of 1552 known compounds mainly sourced from Prestwick Chemical (Illkirch‐Graffenstaden, France), Calbiochem (Merck Millipore), and Selleck Chemicals (Houston, TX, USA). Each compound are resuspended with dimethyl sulfoxide (DMSO) at a concentration of 10 mM, and placed into 96‐well microtiter plates (see Figure 2A for detailed position).

### 2.6 Quantitative real‐time polymerase chain reaction (qPCR)

Total RNA extraction was conducted with the RNeasy Mini Kit (Qiagen, Hilden, Germany), followed by reverse transcription to complementary DNA using the ReverTra Ace qPCR RT Kit (TOYOBO, Osaka, Japan) in accordance with the manufacturer's guidelines. Triplicate qPCR experiments were performed using PowerUp™ SYBR™ Green Master Mix (Thermo Fisher Scientific) on an Applied Biosystems 7300 qPCR system (Thermo Fisher Scientific). Thermal cycling parameters consisted of 95°C for 15 s, 60°C for 30 s, and 72°C for 30 s. Data normalization was achieved by comparing cycle threshold values to the amplified levels of human glyceraldehyde 3‐phosphate dehydrogenase (GAPDH).

The PCR primers used were as follows: AR, F: 5′‐CTTCACCAATGTCAACTCCA‐3′, R: 5′‐TCATTCGGACACACTGGCTG‐3′; KLK3, F: 5′‐CACAGCCTGTTTCATCCTGA‐3′, R: 5′‐AGGTCCATGACCTTCACAGC‐3′; GAPDH, F: 5′‐GAA GGT GAA GGT CGG AGT‐3′, and R: 5′‐GAA GAT GGT GAT GGG ATT TC‐3′.

A GSK‐3β inhibitor AR‐A014418 (Cat no. S7435), an IKK inhibitor BMS‐345541 (Cat no. S8044), and a JAK1/JAK2 inhibitor Momelotinib (CYT387, Cat no. S2219) were purchased from Selleck Chemicals.

### 2.7 Statistical analyses

Mean values along with standard error of the mean (SEM) or standard deviation (SD) are provided for the results. Student's *t*‐test was employed to assess differences between means, with statistical significance defined as a *p*‐value below 0.05. *Z*'‐factor values, a measure of assay robustness, were calculated as described previously: *Z*'‐factor = 1−(3*αp* + 3*αn*)/|*βp*−*βn*|, where *αp* and *αn* denote the standard deviations of positive and negative controls, respectively, and *βp* and *βn* represent the means of positive and negative controls, respectively.^12^ Statistical analyses were conducted using GraphPad Prism version 6 (GraphPad Software, San Diego, CA, USA).

## 3 RESULTS

### 3.1 Application of the AREluc reporter assay in KUCaP13 cells

**FIGURE 1:**
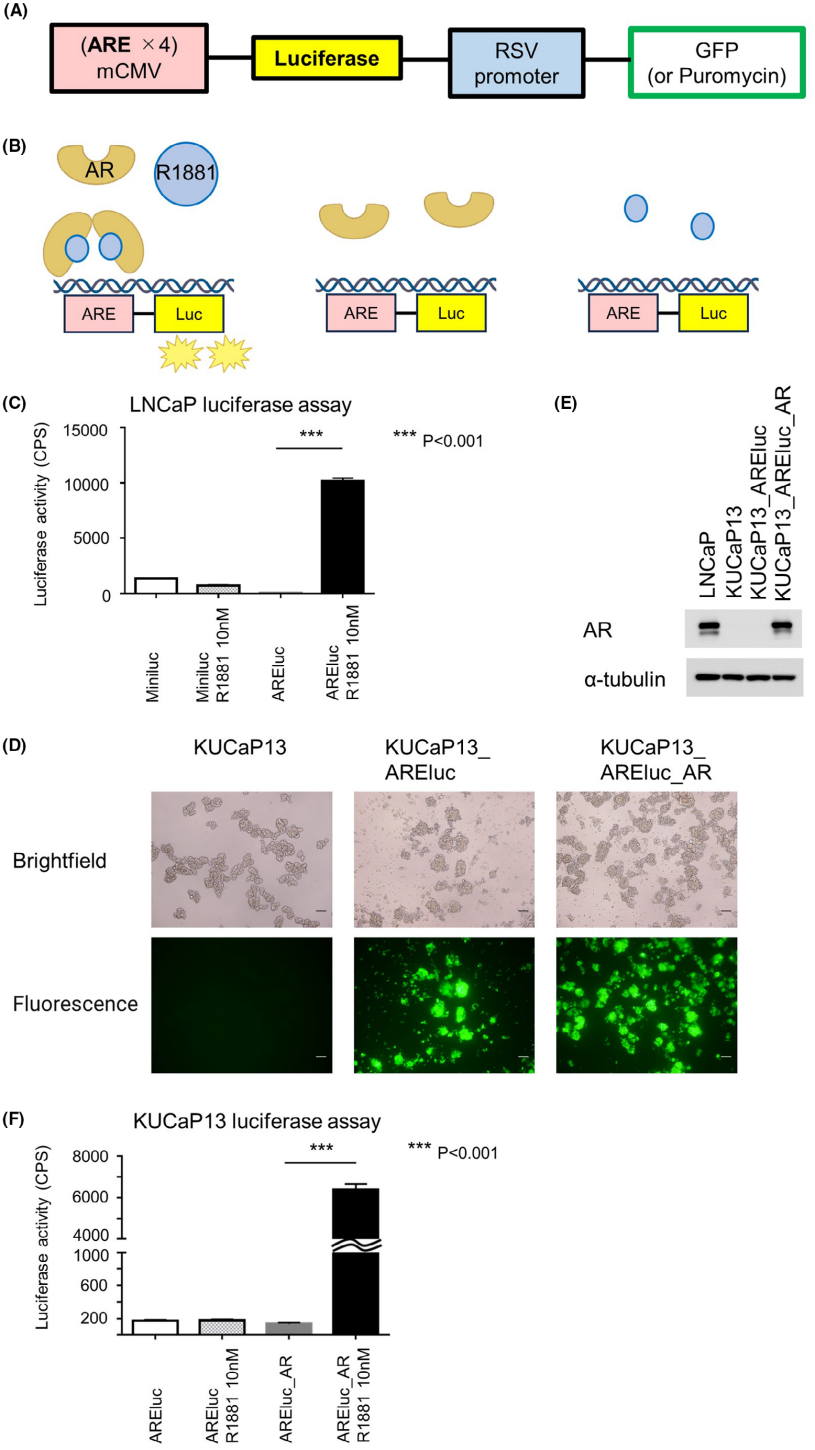
Application of the androgen response element luciferase (AREluc) reporter assay in KUCaP13 cells: (A) The core construction map of the AREluc reporter lentivirus; (B) schema illustrating how the AREluc reporter assay works; (C) validation of the AREluc reporter assay to detect androgen receptor (AR) activity in LNCaP cells. LNCaP_AREluc cells and LNCaP_miniluc cells were incubated with or without 10 nM R1881 for 48 h at 2.0 × 10^4^ cells/well in a white 96‐well plate, followed by evaluation using a luciferase assay. The luciferase activity was measured in absolute counts per second (CPS). The error bars represent the SEM for triplicate measurements. ***p < 0.001; (D) Brightfield and fluorescence microscopic images of KUCaP13 cells, KUCaP13_AREluc cells, and KUCaP13_AREluc_AR cells using a fluorescence microscope. Scale bars indicate 100 μm; (E) western blotting results showing AR expression in KUCaP13_AREluc_AR cells; (F) validation of the AREluc reporter assay in KUCaP13. Both KUCaP13_AREluc cells and KUCaP13_AREluc_AR cells were incubated with ±10 nM R1881 for 72 h at 5.0 × 10^4^ cells/well in a white 96‐well plate, followed by evaluation using a luciferase assay. The luciferase activity was measured in absolute CPS. The error bars represent SEM for quintuplicate (AREluc) or triplicate measurements (AREluc +10 nM R1881, AREluc_AR ± 10 nM R1881). ***p < 0.001.

We developed an AREluc luciferase reporter assay system to detect AR activity using lentivirus. The virus carried a luciferase reporter under a minimal CMV promoter (mCMV) embedded with optimized tandem repeats of the ARE sequence motif (5′‐ TGGAGGAACATATTGTATTTATT) and contained a constitutively expressed GFP selection marker or a puromycin selection marker under the RSV promoter (Figure 1A). When methyltrienolone (R1881), a synthetic androgen agonist, binds to AR, the activated AR then binds to ARE, inducing downstream luciferase expression. The luminescence emitted by luciferase can be easily and rapidly quantified using a luciferase assay. Conversely, AR‐expressing cells, in the absence of R1881, fail to bind to ARE and consequently do not exhibit luciferase expression. Similarly, cells lacking AR expression, despite R1881 addition, do not express luciferase (Figure 1B).

The AR‐expressing prostate cancer cell line, LNCaP, was used to assess AREluc reporter assay functionality. Luminescence was significantly increased in LNCaP_AREluc cells when exposed to R1881 compared with in R1881 absence. In contrast, no such enhancement was observed in LNCaP_miniluc cells (serving as the negative control) upon the addition of R1881 (Figure 1C). These results confirm the AREluc assay's functionality in AR‐expressing cells.

Furthermore, KUCaP13 cells were transduced with the AREluc reporter (containing a GFP selection marker) to generate KUCaP13_ARE‐luc cells. Since KUCaP13 cells lack AR expression, KUCaP13_AREluc_AR cells with forced AR expression served as the positive control in this assay system. GFP signals were detected in both KUCaP13_AREluc cells and KUCaP13_AREluc_AR cells using a fluorescence microscope, confirming successful AREluc transduction (Figure 1D). Western blotting confirmed the expression of AR in KUCaP13_AREluc_AR cells (Figure 1E).

Finally, we validated the functionality of the AREluc reporter assay in KUCaP13 cells. Here, KUCaP13_AREluc_AR cells' luminescence exhibited a significant increase upon exposure to R1881 compared with its absence, whereas no such augmentation was observed in KUCaP13_AREluc cells regardless of the presence or absence of R1881 (Figure 1F). The Z' factor, a quality control parameter calculated for the AREluc reporter assay in KUCaP13, was 0.81, indicating the high performance of the assay.^12^ These results demonstrate the AREluc assay's proper functionality in KUCaP13 cells and its strong applicability in high‐throughput screening.

### 3.2 High‐throughput screening using a chemical library

**FIGURE 2:**
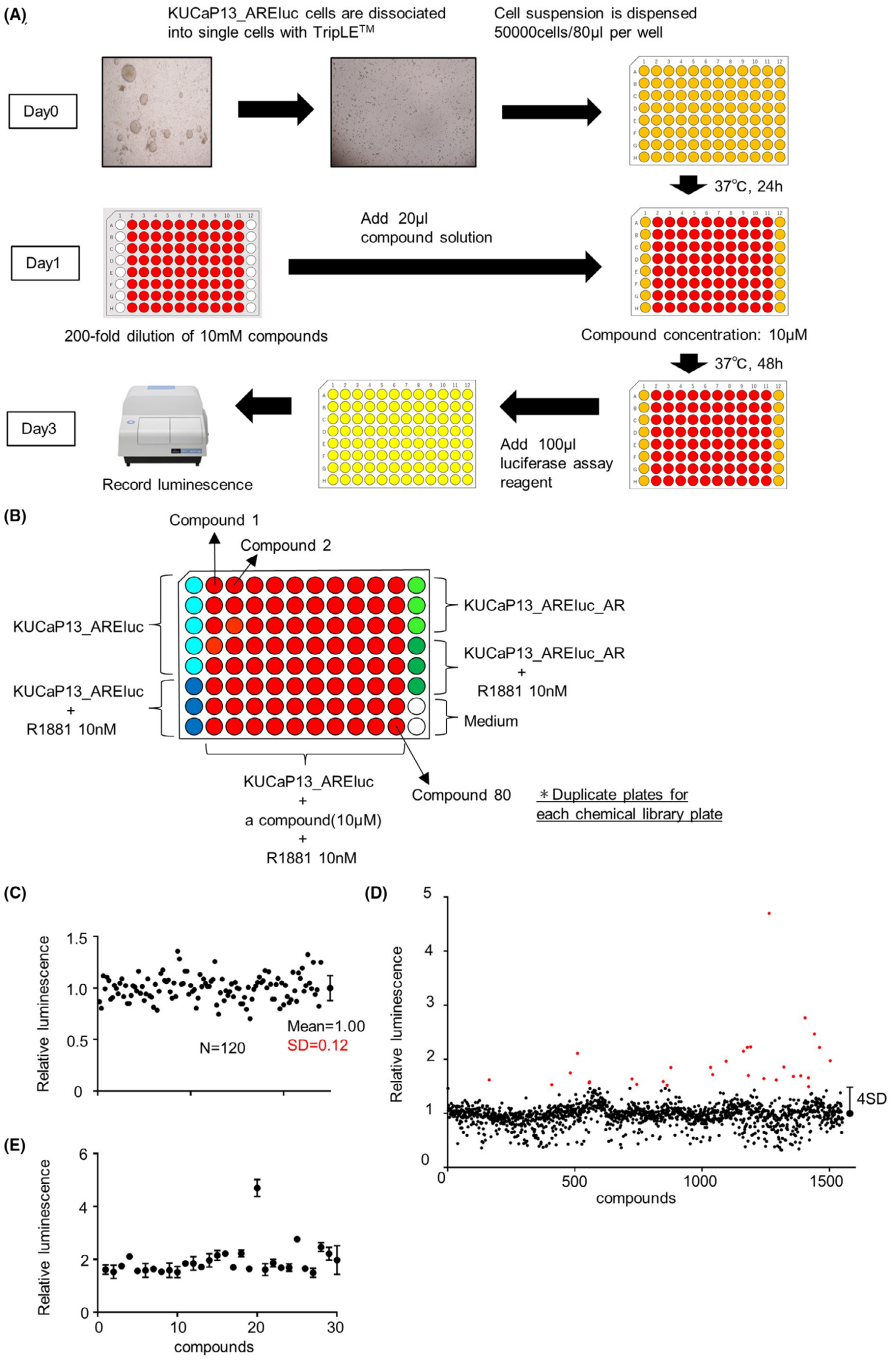
High‐throughput screening using a chemical library: (A) a schematic diagram of the procedure for high‐throughput screening using KUCaP13_AREluc cells; (B) plate layout of high‐throughput screening using a chemical library. For a negative control, KUCaP13_AREluc cells were plated in quintuplicate, and KUCaP13_AREluc cells with 10 nM R1881 were plated in triplicate, according to the positions indicated in the figure. Additionally, to serve as a positive control, KUCaP13_AREluc_AR cells without R1881 and KUCaP13_AREluc_AR cells with 10 nM R1881 were plated in triplicate, adhering to the designated positions shown in the figure. KUCaP13_AREluc cells, treated with 10 μM for each individual compound and supplemented with 10 nM R1881, were positioned in the designated wells of a 96‐well plate, mirroring the arrangement of the chemical library. The wells designated for the negative and positive controls were aligned with a 0.1% DMSO solution, identical to the wells containing the compounds. Duplicate plates were tested for each chemical library plate; (C)–(E) results of the first screening using a chemical library consisting of 1552 known compounds. The relative luminescence of each well was calculated by defining the mean luminescence from the wells of KUCaP13_AREluc cells without R1881 on the same plate as a reference value of 1; (C) Scatter plot of the relative luminescence from the wells of KUCaP13_AREluc cells with 10 nM R1881. The error bar represents mean ± standard deviation (SD); (D) scatter plot of the first screening consisted of 1552 compounds. The mean luminescence of each compound is depicted in the plot. The bar positioned on the far right represents the cutoff value, which is 1 + 4 SD (SD = 0.12). The red dots positioned above 1 + 4 SD (1.48) indicate 30 hit compounds identified in the first screening; (E) scatter plot of 30 hit compounds identified in the first screening. Data represents mean ± SD.

We performed a high‐throughput screening with KUCaP13_AREluc cells using a chemical library, as illustrated in Figure 2A. KUCaP13_AREluc and KUCaP13_AREluc_AR cells were dissociated into single cells using TripLE™ and subsequently seeded (5.0 × 10^4^ cells/80 μL) in white 96‐well plates of androgen‐free medium with or without 10 nM R1881 (Figure 2B), and incubated for 24 h at 37°C. Individual compound solutions (diluted 1:200 in androgen‐free medium) were added (20 μL/well), resulting in a final concentration of 10 μM per compound. After 48 h incubation at 37°C, we added 100 μL of luciferase assay reagent and measured the luminescence. Duplicate plates were tested for each chemical library plate.

The first screening was performed for 1552 compounds of the chemical library by 40 independent measurements using 96‐well plates. The mean ± SD of *Z*' factors in the first screening was 0.87 ± 0.06. In each plate, the relative luminescence of each well was calculated and normalized by the mean luminescence from the wells of KUCaP13_AREluc cells without R1881 on the same plate. The mean relative luminescence values of the wells treated with each compound are listed in Table S1. The SD of the relative luminescence was calculated from the wells of KUCaP13_AREluc cells treated with 10 nM R1881 without any compound, resulting in a value of 0.12 (Figure 2C). We set the cutoff of the relative luminescence value for a significant signal as 1 + 4SD (1.48), identifying 30 hit compounds (Figure 2D). All the hit compounds and their individual results in the first screening are listed in Table 1. Additionally, the scatter plot extracting the relative luminescence of the hit compounds is shown in Figure 2E.

**TABLE 1:** The list of 30 compounds identified in the first screening.

Compound	Relative luminescence		Class
	Mean	SD	
AR‐A014418	4.70	0.23	GSK3β inhibitor
BMS‐345541	2.77	0.06	IkB kinase inhibitor
MK‐571 sodium salt hydrate	2.47	0.12	Leukotriene receptor antagonist
Cyt387	2.23	0.09	Janus kinase inhibitor
A‐966492	2.22	0.04	PARP inhibitor
PD 146176	2.22	0.17	Lipoxygenase inhibitor
Crenolanib	2.15	0.13	FLT/PDGFR inhibitor
Tiabendazole	2.11	0.06	Anthelmintic
BMS 493	1.98	0.38	Inverse pan‐retinoic acid receptor agonist
Anethole‐trithione	1.97	0.18	Bile secretion‐stimulating agent
MTEP hydrochloride	1.86	0.10	Metabotropic glutamate receptor antagonist
Lansoprazole	1.85	0.18	Proton pump inhibitor
Nabumetone	1.85	0.05	NSAID
Tiaprofenic acid	1.75	0.03	NSAID
Methiazole	1.72	0.07	Anthelmintic
OSI‐420	1.70	0.08	Metabolite of erlotinib
PF‐573228	1.70	0.10	Focal adhesion kinase inhibitor
Safinamide mesylate salt	1.69	0.01	Monoamine oxidase inhibitor
Biphenyl‐indanone A	1.66	0.01	Metabotropic glutamate receptor potentiator
Fenobam	1.65	0.02	Metabotropic glutamate receptor antagonist
Demeclocycline hydrochloride	1.64	0.05	Antibiotic
Riluzole hydrochloride	1.62	0.12	GABA uptake inhibitor
SB 204741	1.62	0.16	Serotonin receptor antagonist
Rabeprazole Sodium salt	1.59	0.19	Proton pump inhibitor
Tenatoprazole	1.59	0.18	Proton pump inhibitor
Dacarbazine	1.57	0.08	Antineoplastic alkylating agent
Leflunomide	1.54	0.02	DMARD
Erlotinib	1.53	0.18	EGFR inhibitor
Butylparaben	1.52	0.15	Antimicrobial agent
LY255582	1.50	0.12	Opioid receptor antagonist

Abbreviations: DMARD, disease‐modifying antirheumatic drug; EGFR, epidermal growth factor receptor; FLT, FMS‐like tyrosine kinase; GABA, gamma‐aminobutyric acid; GSK3β, glycogen synthase kinase 3β; NSAID, non‐steroidal anti‐inflammatory drug; PARP, poly ADP ribose polymerase; PDGFR, platelet‐derived growth factor receptor.

### 3.3 Validation of hit compounds

**FIGURE 3:**
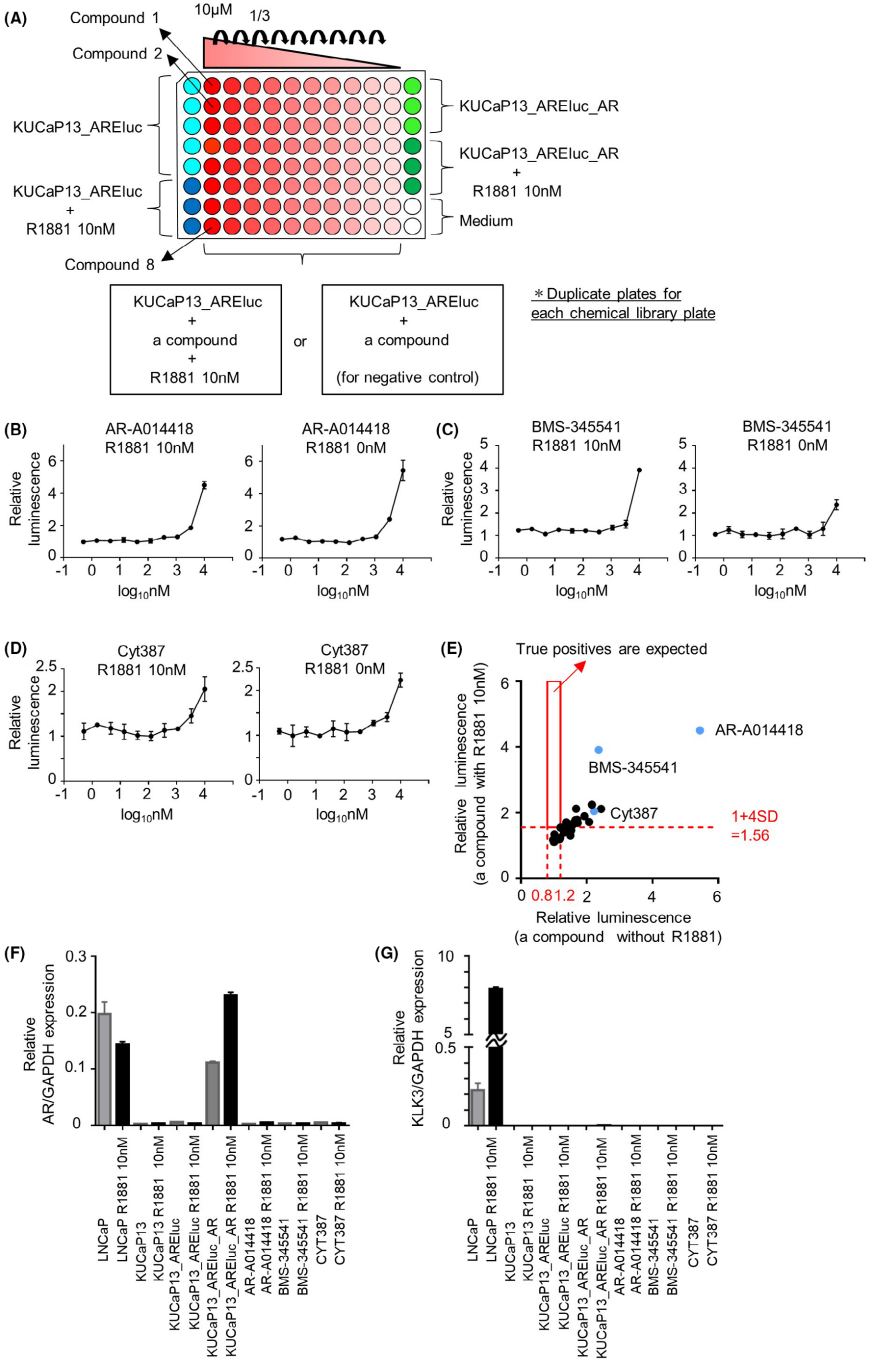
Validation of hit compounds: (A) plate layout of the second screening. The negative and positive control wells, located in either end row of the plate, were prepared in the same manner as in the first screening. KUCaP13_AREluc cells, treated with a 1:3 serial dilution series of eight compounds starting at a final concentration of 10 μM, were plated, adhering to the designated positions shown in the figure. A 10 nM R1881 was added to the wells containing the compound, while it was not added to the wells of the negative control plates. Duplicate plates were tested for each plate; (B)–(D) results of three representative compounds in the second screening; (B) AR‐A014418; (C) BMS‐345541; (D) Cyt387. Data represents mean ± standard deviation (SD). The graph on the left is with R1881, and the graph on the right is without R1881; (E) the scatter plot depicts the results of the second screening. For each compound, a plot was created with the maximum relative luminescence value of the wells with R1881 on the *y*‐axis and the relative luminescence value of the wells without R1881 at the concentrations that exhibited the maximum values with R1881 on the *x*‐axis. The red line on the y‐axis represents the cutoff value of relative luminescence from wells with R1881 added to the compound, set at 1 + 4 SD (SD = 0.14), or 1.56. The relative luminescence from wells without R1881 added to the compound was expected to fall between the two red lines, 0.8 and 1.2. The region enclosed by the solid red line is the area where true positives are expected. The compounds shown in (B)–(D) were plotted in blue; (F, G) mRNA expression levels of *AR* (F) and *KLK3* (G) normalized by the expression of GAPDH using quantitative qPCR. All cell types were cultured with or without 10 nM R1881. After 24 h, LNCaP cells, KUCaP13 cells, and KUCaP13_AREluc_AR cells were treated with 0.1% DMSO and cultured for a total of 72 h. In addition, KUCaP13_AREluc cells were treated with 0.1% DMSO, or 10 μM of AR‐A014418, BMS‐345541, and CYT387 after 24 h, and cultured for a total of 72 h. Data represent mean ± standard error of the mean.

We conducted a second screening using luciferase assay to validate the 30 hit compounds and eliminate false positives. False positives were detected by comparing the plates with and without R1881 added to the wells containing each hit compound. The screening was performed in duplicates. The second screening was performed in the same manner as the first screening (Figure 2A). As shown in Figure 3A, in a 96‐well plate, eight compounds were initially diluted to 50 μM and further diluted in a threefold serial manner to prepare a dilution series. Then, 20 μL of each compound solution was added to the 96‐well white plates with seeded cells, resulting in a 3‐fold serial dilution series starting at a final concentration of 10 μM for each of the eight compounds.

The second screening was performed for the 30 hit compounds by 16 individual measurements using 96‐well plates. The mean ± SD of Z' factors in the second screening was 0.81 ± 0.09. The results of each plate were standardized in the same manner as in the first screening. The results of the second screening are shown in Figure 3B–D and Figure S2. For all candidate compounds, the increase in relative luminescence was observed with or without R1881 addition, exhibiting similar dose–response curves. The relative luminescence cutoff value from wells with R1881 addition was determined to be 1 + 4 SD (1.56), using a similar calculation as in the first screening. The relative luminescence from wells without R1881 addition (serving as the negative control) was expected to fall within the range of 0.8–1.2 if there was no signal. Notably, none of the compounds met both criteria (increased luminescence only with R1881 and control range without R1881) for true positives (Figure 3E).

We also checked the mRNA expression of AR and its downstream target *KLK3* induced by the hit compounds for three representative candidate compounds: AR‐A014418 (a GSK‐3β inhibitor), BMS‐345541 (an IKK inhibitor), and CYT387 (a JAK inhibitor) using qPCR. However, these drugs did not alter the expression of *AR* or *KLK3* (Figure 3F,G). Notably, *KLK3* expression was also not induced in KUCaP13_AREluc_AR cells with overexpressed AR, suggesting that AR expression alone is not sufficient to activate the AR pathway in KUCaP13 cells. In summary, the compounds identified as candidate hits in the first screening underwent validation through luciferase assay and qPCR. However, all these compounds were considered false positives. No compound achieved AR re‐expression in the KUCaP13 cells.

## 4 DISCUSSION

NEPC is an aggressive variant of prostate cancer. However, due to its rarity and the lack of experimental models, its biology has remained unclear. Recent advances in genomic analysis of human specimens have revealed NEPC's genomic and molecular biological characteristics. The acquisition of *MYCN* oncogenes, coupled with the loss of tumor suppressor genes (*TP53*, *RB1*, and *PTEN*), play a critical role in NEPC development.^13^, ^14^ However, since these genes are not direct therapeutic targets, NEPC remains a cancer with a very poor prognosis for which no effective treatment currently exists. The origin of NEPC, whether it arises from a small subset of pre‐existing NE cells in the prostate gland or from the transdifferentiation of adenocarcinoma cells, has sparked controversy.^15^, ^16^ However, recent reports strongly suggest that t‐NEPC undergoes transdifferentiation from an adenocarcinoma due to epithelial plasticity.^6^, ^17^, ^18^ The mechanism underlying lineage plasticity in t‐NEPC is thought to be epigenetic and potentially reversible; however, the detailed processes have not been fully clarified.^4^

Despite recent advances in t‐NEPC research, no t‐NEPC cell lines are available for genetic manipulation or large‐scale compound screening. The solitary widely employed cell line derived from a patient with NEPC is NCI‐H660. Initially classified as originating from small‐cell lung cancer, it was subsequently recognized as prostatic in nature due to the presence of *TMPRSS2*‐*ERG* fusion.^19^ However, since the cell line originated from a lymph node metastasis in a patient who had died within a month of diagnosis without receiving treatment, NCI‐H660 is likely to be a de novo NEPC, and it is uncertain whether it originally expressed AR.^19^, ^20^ Therefore, NCI‐H660 is not a suitable model for studying the lineage plasticity of t‐NEPC. In contrast, a novel t‐NEPC cell line, named KUCaP13, was created from the tissue of a patient initially diagnosed with adenocarcinoma, which subsequently transformed into NEPC. KUCaP13 is the initial patient‐derived t‐NEPC cell line displaying the triple loss of tumor suppressors crucial for NEPC progression via lineage plasticity.^10^ In prostate cancer, lineage alteration can be evaluated by AR activity, unlike in other cancer types, such as lung cancer and melanoma. Hence, in the present study, we focused on identifying compounds that can re‐express AR through chemical screening using KUCaP13 cells. Identifying these compounds will contribute to understanding the mechanism of lineage plasticity in t‐NEPC and enable the development of innovative therapies for t‐NEPC in combination with ARSIs.

AR is crucial in the development and progression of most prostate cancer cases. AR, a ligand‐dependent transcription factor, is localized in the cytoplasm when inactive.^21^ Ligand binding, by the native androgens testosterone and 5α‐dihydrotestosterone, induces nuclear translocation and receptor dimerization. In the nucleus, AR recruits various collaborative factors and binds ARE sequences located in the enhancer and promoter regions of target genes. Thus, it regulates the transcription of androgen‐responsive genes, including prostate‐specific antigens.^22^ Azeem et al. developed ARE‐based assays that can test AR activity on a large scale, rendering them valuable for drug screening.^23^ Luciferase is frequently employed as a reporter in high‐throughput screening assays due to its remarkable sensitivity, extensive dynamic range, and swift measurement capabilities.^24^ Thus, in our study, a reporter gene, AREluc, was transduced into KUCaP13 cells to detect AR activity using luciferase assay. The positive control cells, KUCaP13_AREluc overexpressing AR, exhibited enhanced luminescence upon the administration of synthetic androgens. This reporter assay system enables the assessment of plasticity reversibility by detecting AR expression in KUCaP13 and facilitates high‐throughput screenings. This is a novel screening system for evaluating the reversibility of plasticity using the t‐NEPC cell line.

Compounds responsible for AR re‐expression in KUCaP13 cells were screened using the library of known compounds provided by our Drug Discovery Research. Previously, we identified disulfiram as a sensitizer of cisplatin in bladder cancer through high‐throughput chemical screening using this library.^25^ Our present study detected compounds that enhance luciferase luminescence and identified 30 hit compounds in the first screening. However, all hit compounds were confirmed as false positives through the second screening and qPCR. One possible reason is the inhibitory effect of the compounds on luciferase. In firefly luciferase reporter gene assays, inhibitors of luciferase can act intracellularly to prolong the lifespan of ectopically expressed firefly luciferase enzyme. This leads to heightened luciferase activity, which can be visually indistinguishable from the activation of reporter gene transcription.^26^ In previous studies, inhibitory activity against luciferase was identified in 12% of the compounds present in a chemical library.^27^ In our present study, 73% of the compounds that exhibited false positives shared the same root scaffolds as luciferase inhibitors that had been reported previously.^27^

The reversibility of lineage plasticity in t‐NEPC has been demonstrated in gene‐engineered mice, but this has not been confirmed in models from clinical samples. Recently, the reversibility of lineage plasticity in castration‐resistant prostate cancer (CRPC) in the intermediate/lineage‐plastic state through the JAK/STAT pathway has been reported.^28^ The combination of a JAK inhibitor and an FGFR inhibitor increased AR expression in organoids with enhanced JAK/STAT signaling from patients with CRPC but did not affect AR expression in organoids from patients with NEPC.^28^ In the current situation, where the reversibility of cellular lineage of t‐NEPC in clinical specimens has not been demonstrated, the establishment of a screening system to detect AR re‐expression using t‐NEPC cell lines and applying it in large‐scale chemical screening may open a door for an innovative development.

In our study, a single compound alone was unable to re‐express AR in KUCaP13 cells. However, since lineage plasticity involves multiple processes, there may be potential for AR re‐expression in t‐NEPC utilizing a synergistic combination of multiple compounds. Alternatively, it may be that the regulation of cellular lineage in cells that had undergone complete transdifferentiation to t‐NEPC is challenging. In a previous study identifying the role of JAK/STAT signaling in NEPC transdifferentiation, it was reported that therapeutic timing was critical due to cell‐state heterogeneity in a patient with CRPC and the absence of JAK/STAT activation in NEPC.^28^ Hence, reversing the cell lineage in the earlier stages of transdifferentiation may be crucial.

Our study has several limitations. This screening system was challenged by the absence of a compound that can serve as a positive control. Before compound screening, we independently tested several drugs that could potentially impact plasticity in t‐NEPC, including an EZH2 inhibitor (EPZ‐6438), a bromodomain inhibitor ((+)‐JQ1), an LSD inhibitor (GSK2879552 2HCl), and a histone deacetylase inhibitor (Trichostatin A); however, none of them elicited an increase in luminescence in the presence of R1881 (data not shown). Therefore, we established KUCaP13_AREluc_AR cells as the positive control. Furthermore, owing to technical limitations associated with the nature of KUCaP13 cell growth in suspension and the difficulty of refreshing culture media during the compound screening process, we measured luminescence 2 days post‐exposure to the compounds. In a previous report using organoids, human organoids had been exposed to compounds for 14 days to assess the reversibility of plasticity.^28^ In human prostate cancer‐derived cells, it may take more than 2 days for AR re‐expression. We set the entire screening to be completed in 3 days since luminescence in KUCaP13_AREluc_AR cells showed the most significant increase after exposure to R1881 cells for a 3‐day incubation period (data not shown). Additionally, as cell viability was not assessed, it is possible that some of the compounds could have caused cell death at 10 μM, potentially resulting in false negatives.

In conclusion, we developed a highly sensitive screening system to evaluate the reversibility of plasticity in t‐NEPC using KUCaP13. Despite not achieving the goal of AR‐re‐expression, this study paves the way for the application of high‐throughput screening approaches in t‐NEPC research and future exploration of alternative strategies, including targeting earlier stages of transdifferentiation or investigating synergistic combinations of compounds.

## AUTHOR CONTRIBUTIONS

**Tomohiro Fukui:** Data curation (lead); formal analysis (lead); funding acquisition (equal); investigation (lead); methodology (equal); validation (lead); visualization (lead); writing – original draft (lead); writing – review and editing (equal). **Kosuke Okasho:** Investigation (equal); resources (lead); writing – review and editing (equal). **Yukiko Okuno:** Formal analysis (equal); resources (equal); visualization (equal); writing – review and editing (equal). **Maki Fujiwara:** Investigation (equal). **Kensuke Hikami:** Investigation (equal). **Arinobu Fukunaga:** Investigation (equal). **Takuro Sunada:** Formal analysis (equal); investigation (equal). **Yuki Kita:** Formal analysis (equal); writing – review and editing (equal). **Takayuki Sumiyoshi:** Formal analysis (equal); writing – review and editing (equal). **Takayuki Goto:** Formal analysis (equal); writing – review and editing (equal). **Ryoichi Saito:** Formal analysis (equal); writing – review and editing (equal). **Osamu Ogawa:** Project administration (equal); supervision (equal). **Takashi Kobayashi:** Project administration (equal); resources (equal); supervision (equal). **Shusuke Akamatsu:** Conceptualization (lead); formal analysis (equal); funding acquisition (lead); methodology (equal); project administration (lead); supervision (lead); writing – review and editing (lead).

## FUNDING INFORMATION

This research was funded by JSPS KAKENHI Grant Number JP21K19568 to Shusuke Akamatsu and by the funding from the Japanese Urological Association to Shusuke Akamatsu. Partial financial support was received from Bayer Yakuhin, Ltd to Tomohiro Fukui.

## CONFLICT OF INTEREST STATEMENT

Tomohiro Fukui received research funding from Bayer Yakuhin, Ltd.

## Supporting information


**Figure S1.** A schematic diagram of detailing the treatment‐related neuroendocrine prostate cancer (t‐NEPC) characteristics of KUCaP13 and outlining the experimental method.
We established a novel t‐NEPC cell line, KUCaP13, derived from patient‐derived xenograft (PDX) that originated from the tissue of a patient initially diagnosed with adenocarcinoma which later recurred as NEPC. HE staining showed that the patient’s original tumor tissue was diagnosed as small cell carcinoma. Transcriptome analysis using unsupervised clustering by androgen receptor (AR) pathway genes and NEPC‐related genes showed a clear distinction between adenocarcinoma and NEPC, with both KUCaP13 PDX and cell line clustering with NEPC.
Our objective was to demonstrate the reversibility of lineage plasticity in t‐NEPC through drug screening with KUCaP13. We transduced a reporter gene, AREluc, into KUCaP13 using lentivirus to detect AR activity. We conducted chemical screening in an attempt to identify hit compounds responsible for the re‐expression of AR in KUCaP13.
**Figure S2.** Results of the second screening. (A)–(AA) graphs indicate results for 27 compounds other than the three compounds shown in Figure 3. Data represent mean ± standard deviation (SD). The graph on the left is with R1881, and the graph on the right is without R1881.

**Table S1.** The list of 1552 compounds and the relative luminescence in the first screening.

## Data Availability

Please contact the corresponding author for all the data in the current study if needed.
