# Supplementary figures and images for: A highly sensitive screening system to evaluate the reversibility of neuroendocrine prostate cancer to prostate adenocarcinoma
Source: Cancer Med. 2025 Feb 27;14(5):e70047. doi: 10.1002/cam4.70047 (PMC11865886; doi:10.1002/cam4.70047)

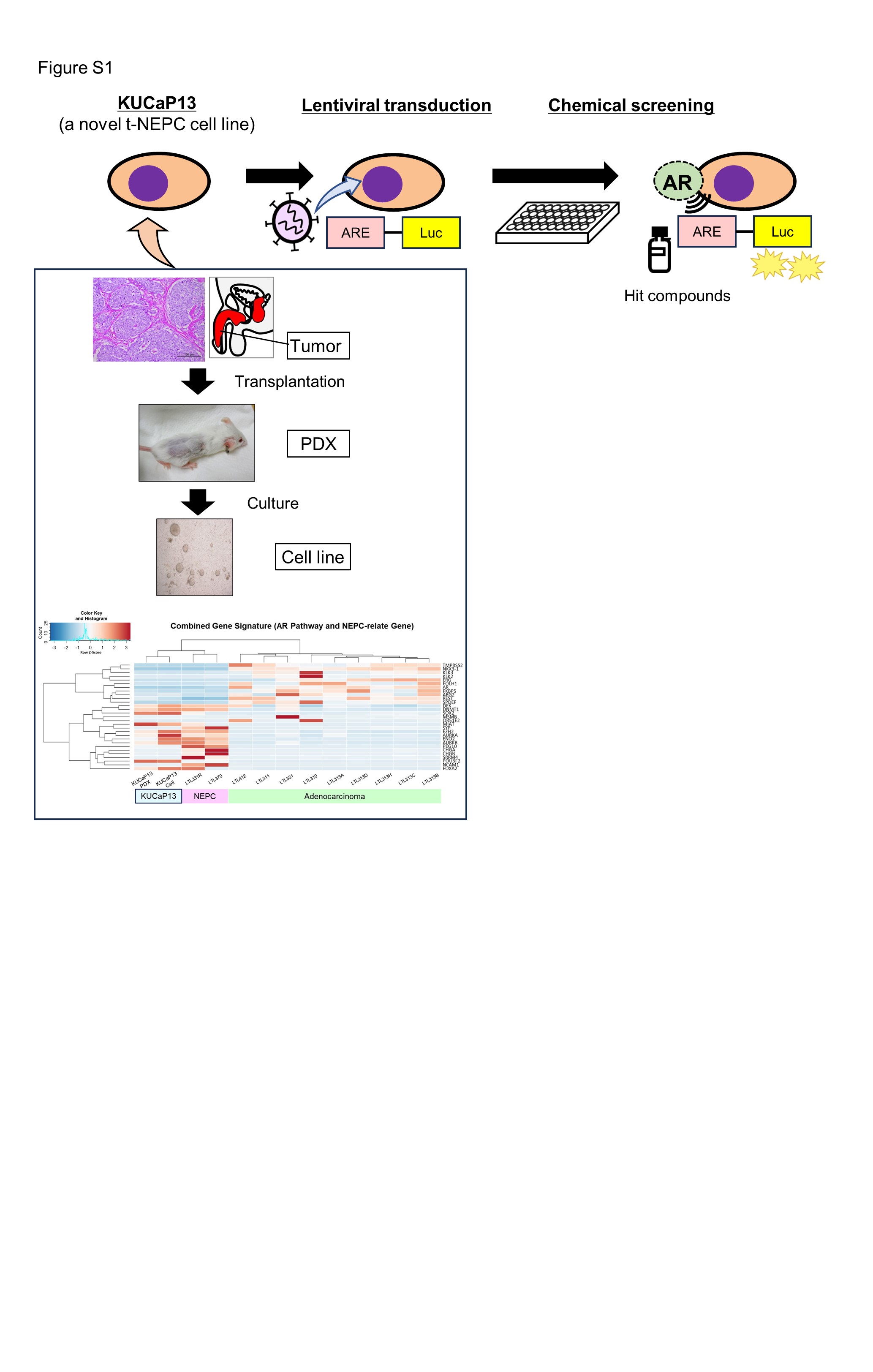

Supplement: Supplementary file 1 — Figure S1. A schematic diagram of detailing the treatment‐related neuroendocrine prostate cancer (t‐NEPC) characteristics of KUCaP13 and outlining the experimental method. We established a novel t‐NEPC cell line, KUCaP13, derived from patient‐derived xenograft (PDX) that originated from the tissue of a patient initially diagnosed with adenocarcinoma which later recurred as NEPC. HE staining showed that the patient’s original tumor tissue was diagnosed as small cell carcinoma. Transcriptome analysis using unsupervised clustering by androgen receptor (AR) pathway genes and NEPC‐related genes showed a clear distinction between adenocarcinoma and NEPC, with both KUCaP13 PDX and cell line clustering with NEPC. Our objective was to demonstrate the reversibility of lineage plasticity in t‐NEPC through drug screening with KUCaP13. We transduced a reporter gene, AREluc, into KUCaP13 using lentivirus to detect AR activity. We conducted chemical screening in an attempt to identify hit compounds responsible for the re‐expression of AR in KUCaP13. Figure S2. Results of the second screening. (A)–(AA) graphs indicate results for 27 compounds other than the three compounds shown in Figure 3. Data represent mean ± standard deviation (SD). The graph on the left is with R1881, and the graph on the right is without R1881. [file CAM4-14-e70047-s001.zip › FigureS1.jpg]

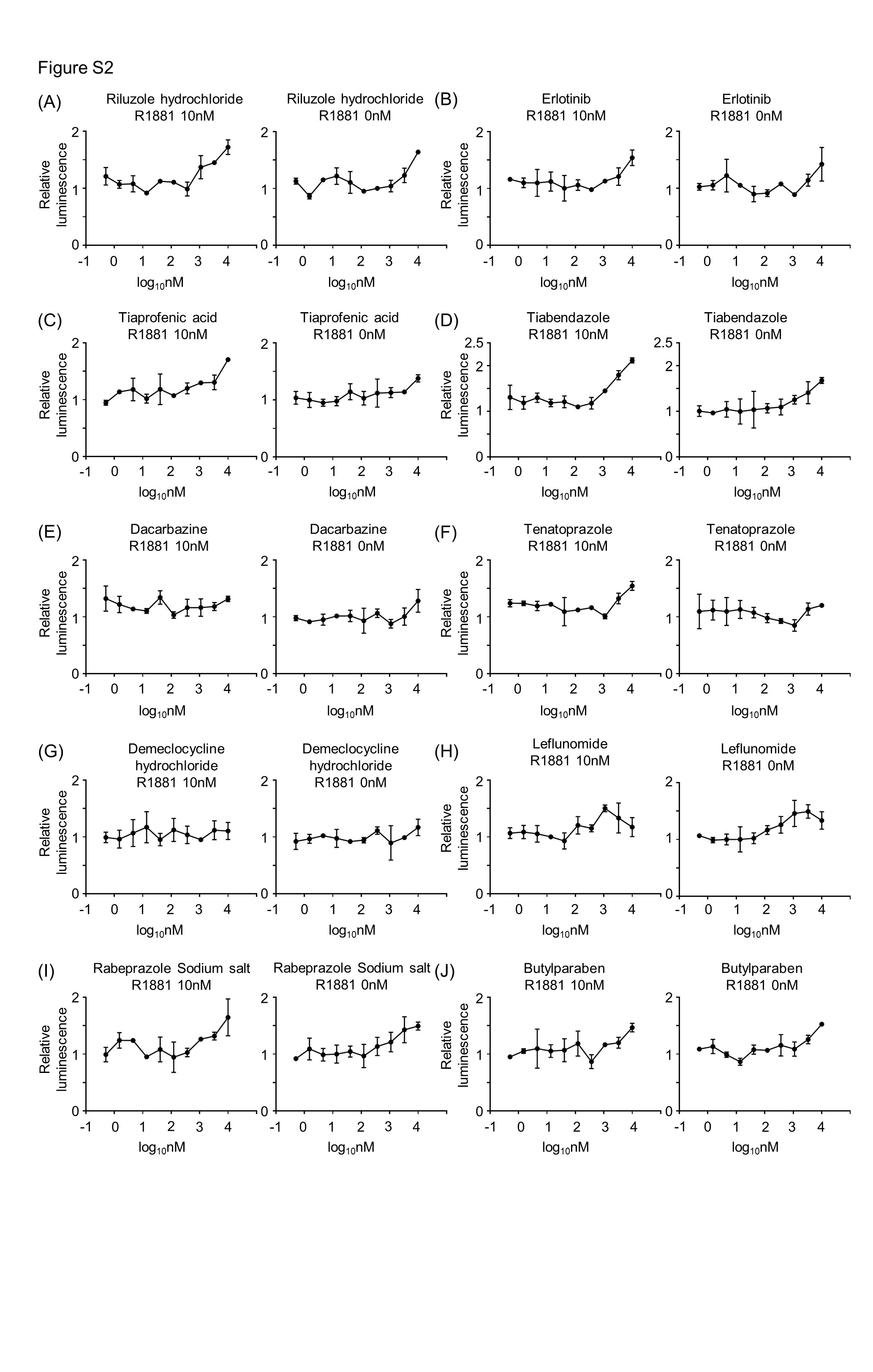

Supplement: Supplementary file 1 — Figure S1. A schematic diagram of detailing the treatment‐related neuroendocrine prostate cancer (t‐NEPC) characteristics of KUCaP13 and outlining the experimental method. We established a novel t‐NEPC cell line, KUCaP13, derived from patient‐derived xenograft (PDX) that originated from the tissue of a patient initially diagnosed with adenocarcinoma which later recurred as NEPC. HE staining showed that the patient’s original tumor tissue was diagnosed as small cell carcinoma. Transcriptome analysis using unsupervised clustering by androgen receptor (AR) pathway genes and NEPC‐related genes showed a clear distinction between adenocarcinoma and NEPC, with both KUCaP13 PDX and cell line clustering with NEPC. Our objective was to demonstrate the reversibility of lineage plasticity in t‐NEPC through drug screening with KUCaP13. We transduced a reporter gene, AREluc, into KUCaP13 using lentivirus to detect AR activity. We conducted chemical screening in an attempt to identify hit compounds responsible for the re‐expression of AR in KUCaP13. Figure S2. Results of the second screening. (A)–(AA) graphs indicate results for 27 compounds other than the three compounds shown in Figure 3. Data represent mean ± standard deviation (SD). The graph on the left is with R1881, and the graph on the right is without R1881. [file CAM4-14-e70047-s001.zip › FigureS2_1.jpg]

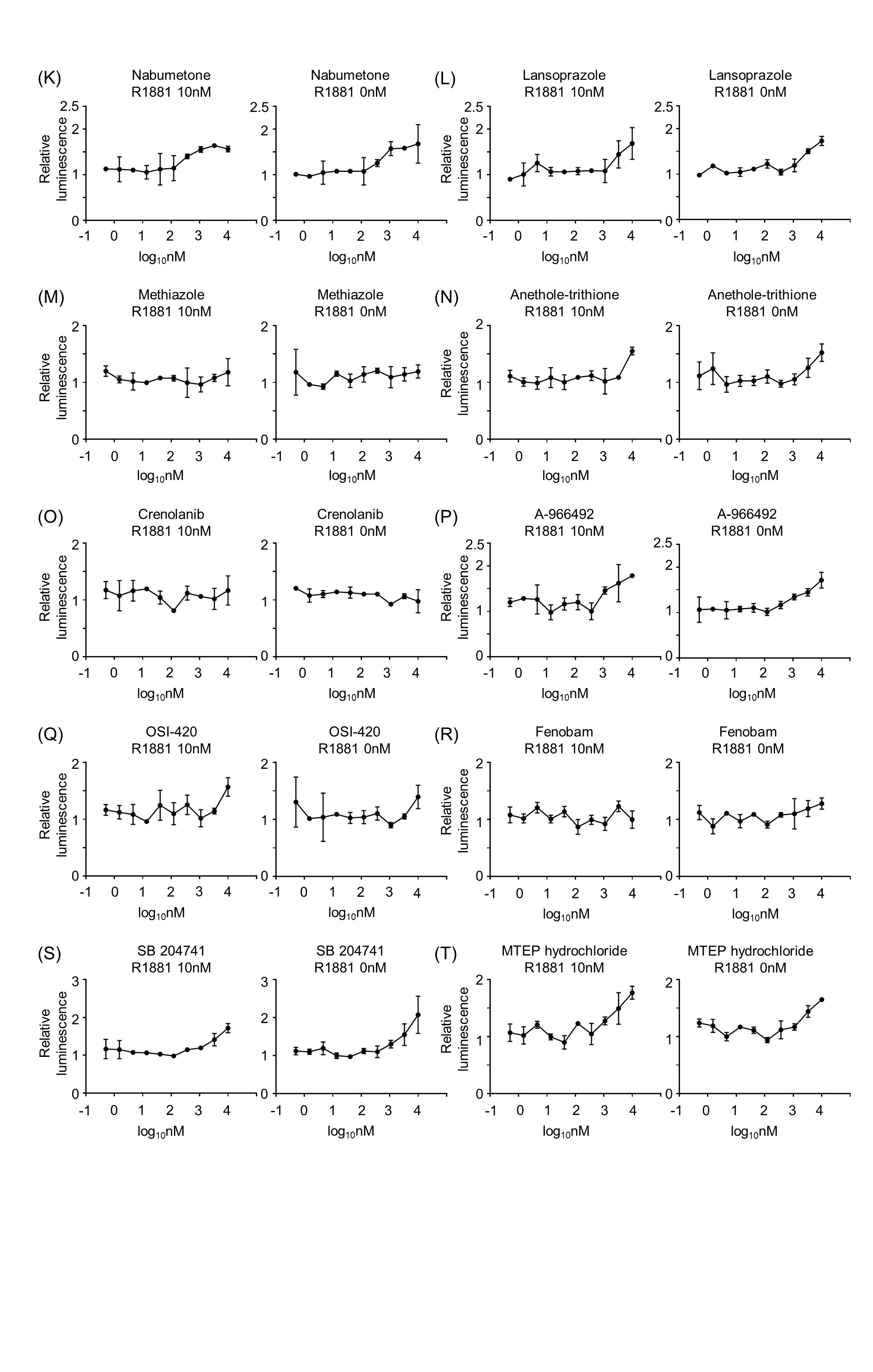

Supplement: Supplementary file 1 — Figure S1. A schematic diagram of detailing the treatment‐related neuroendocrine prostate cancer (t‐NEPC) characteristics of KUCaP13 and outlining the experimental method. We established a novel t‐NEPC cell line, KUCaP13, derived from patient‐derived xenograft (PDX) that originated from the tissue of a patient initially diagnosed with adenocarcinoma which later recurred as NEPC. HE staining showed that the patient’s original tumor tissue was diagnosed as small cell carcinoma. Transcriptome analysis using unsupervised clustering by androgen receptor (AR) pathway genes and NEPC‐related genes showed a clear distinction between adenocarcinoma and NEPC, with both KUCaP13 PDX and cell line clustering with NEPC. Our objective was to demonstrate the reversibility of lineage plasticity in t‐NEPC through drug screening with KUCaP13. We transduced a reporter gene, AREluc, into KUCaP13 using lentivirus to detect AR activity. We conducted chemical screening in an attempt to identify hit compounds responsible for the re‐expression of AR in KUCaP13. Figure S2. Results of the second screening. (A)–(AA) graphs indicate results for 27 compounds other than the three compounds shown in Figure 3. Data represent mean ± standard deviation (SD). The graph on the left is with R1881, and the graph on the right is without R1881. [file CAM4-14-e70047-s001.zip › FigureS2_2.jpg]

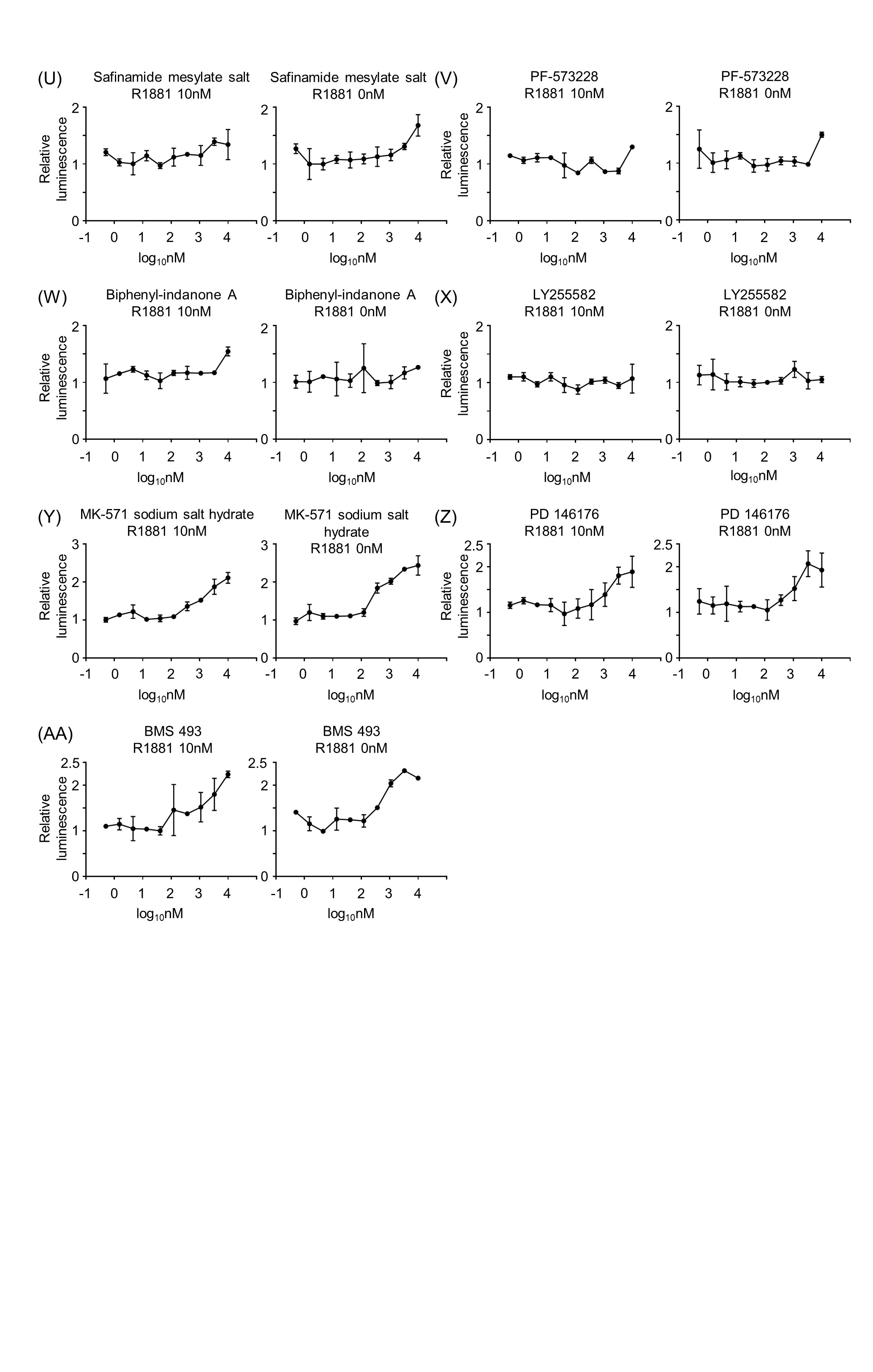

Supplement: Supplementary file 1 — Figure S1. A schematic diagram of detailing the treatment‐related neuroendocrine prostate cancer (t‐NEPC) characteristics of KUCaP13 and outlining the experimental method. We established a novel t‐NEPC cell line, KUCaP13, derived from patient‐derived xenograft (PDX) that originated from the tissue of a patient initially diagnosed with adenocarcinoma which later recurred as NEPC. HE staining showed that the patient’s original tumor tissue was diagnosed as small cell carcinoma. Transcriptome analysis using unsupervised clustering by androgen receptor (AR) pathway genes and NEPC‐related genes showed a clear distinction between adenocarcinoma and NEPC, with both KUCaP13 PDX and cell line clustering with NEPC. Our objective was to demonstrate the reversibility of lineage plasticity in t‐NEPC through drug screening with KUCaP13. We transduced a reporter gene, AREluc, into KUCaP13 using lentivirus to detect AR activity. We conducted chemical screening in an attempt to identify hit compounds responsible for the re‐expression of AR in KUCaP13. Figure S2. Results of the second screening. (A)–(AA) graphs indicate results for 27 compounds other than the three compounds shown in Figure 3. Data represent mean ± standard deviation (SD). The graph on the left is with R1881, and the graph on the right is without R1881. [file CAM4-14-e70047-s001.zip › FigureS2_3.jpg]
